# GLP-1 Receptor Agonists in Cardiac Surgery: From Metabolic Drug to Potential Perioperative Cardioprotective Agent

**DOI:** 10.3390/jcdd13070305

**Published:** 2026-07-03

**Authors:** Vasiliki Androutsopoulou, Vanesa Brecher, Andrew Xanthopoulos, Dimitrios V. Avgerinos, Thanos Athanasiou, Dimitrios E. Magouliotis

**Affiliations:** 1 Department of Cardiothoracic Surgery, Faculty of Medicine, University of Thessaly, 41110 Larissa, Greece; androutsopoulouvasiliki@uth.gr; 2 Department of Cardiac Surgery Research, Lankenau Institute for Medical Research, Wynnewood, PA 19096, USA; vb4075@pcom.edu; 3 Department of Cardiology, Faculty of Medicine, University of Thessaly, 41110 Larissa, Greece; andrewvxanth@gmail.com; 4 Department of Cardiac Surgery, Onassis Cardiac Surgery Center, 17674 Athens, Greece; davgerinos@gmail.com; 5 Department of Surgery and Cancer, Imperial College London, London SW7 2AZ, UK; t.athanasiou@imperial.ac.uk

**Keywords:** GLP-1 receptor agonists, semaglutide, liraglutide, cardiac surgery, cardioprotection, perioperative management, postoperative atrial fibrillation, ischemia–reperfusion injury, coronary artery bypass grafting, TAVR

## Abstract

Glucagon-like peptide-1 receptor agonists (GLP-1 RAs) have rapidly evolved from glucose-lowering agents to central players in cardiovascular risk reduction. Evidence from landmark randomized controlled trials has established their capacity to reduce major adverse cardiovascular events, promote anti-inflammatory signaling, attenuate ischemia–reperfusion injury, and improve myocardial metabolic efficiency. As the prevalence of obesity, type 2 diabetes mellitus, and heart failure in the cardiac surgical population grows, GLP-1 RAs are increasingly encountered in the perioperative setting. Yet the cardiac surgery literature has yet to synthesize their emergent role coherently. This is a narrative review; no systematic review or meta-analysis was performed. This narrative review integrates mechanistic, clinical, and translational evidence to reframe GLP-1 RAs as potential perioperative cardioprotective agents in patients undergoing cardiac surgery. We examine receptor-level biology, evidence from the GLOBE randomized trial, observational data linking GLP-1 RA use to reduced postoperative atrial fibrillation after coronary artery bypass grafting, the rationale for the forthcoming REVERSE-TAVR trial, and evolving perioperative management guidelines. Key evidence gaps are identified, including the absence of prospective data in open cardiac surgery, aortic surgery, and high-acuity populations. We propose a research agenda and conceptual framework to guide future investigation into GLP-1 RAs as a new dimension of perioperative cardioprotection. The current evidence is hypothesis-generating; a definitive perioperative cardioprotective benefit has not yet been demonstrated in cardiac surgery populations, and these agents are presented here as potential rather than proven cardioprotective tools.

## 1. Introduction

Glucagon-like peptide-1 receptor agonists (GLP-1 RAs) represent one of the most consequential pharmacological advances of the past two decades. First introduced as incretin-based glucose-lowering agents for type 2 diabetes mellitus (T2DM), this drug class has undergone a profound conceptual transformation: it is now understood as a systemic cardiometabolic intervention with effects on myocardial energetics, vascular inflammation, autonomic tone, and organ protection that extend far beyond any glycemic action [1,2].

The cardiovascular outcome trial era, launched by the LEADER trial in 2016 and culminating in the SELECT trial in 2023, established a consistent signal of major adverse cardiovascular event (MACE) reduction across GLP-1 RA agents, patient phenotypes, and levels of baseline cardiovascular risk [3]. These findings prompted the European Society of Cardiology and the American Diabetes Association to formally integrate GLP-1 RAs into cardiovascular risk reduction algorithms [4,5].

Despite this trajectory, the existing GLP-1 RA literature has developed almost entirely within the domain of ambulatory cardiovascular medicine. The cardiac surgery population has remained a systematic blind spot. This is paradoxical, because the phenotype of the contemporary cardiac surgery patient overlaps substantially with those in whom GLP-1 RAs have demonstrated the most pronounced benefit. Patients presenting for coronary artery bypass grafting (CABG), surgical aortic valve replacement, or complex aortic repair increasingly carry a constellation of obesity, T2DM, heart failure with preserved ejection fraction (HFpEF), and chronic systemic inflammation [6]. Beyond comorbidity overlap, the physiological demands of cardiac surgery with cardiopulmonary bypass introduce an acute cardiometabolic stress environment characterized by global myocardial ischemia–reperfusion (I-R) injury, sterile systemic inflammation, perioperative hyperglycemia, and hemodynamic instability. These are precisely the biological axes on which GLP-1 RAs exert their most mechanistically well-characterized effects. Yet the cardiac surgery literature has historically approached these agents solely as glycemic drugs whose gastric motility effects necessitate cautious preoperative discontinuation, rather than as potential perioperative cardioprotective tools [7].

The timing of this review is deliberate and justified by a specific cluster of developments that postdate the existing literature. A 2025 scoping review of GLP-1 RA use in cardiac procedures found only three qualifying studies as of April 2024, all evaluating daily dosed liraglutide with glycemic control as the primary lens [8]. Since that review, three developments have materially changed the landscape. First, direct human cardiomyocyte data published in 2025 demonstrated that semaglutide reduces late sodium current and sarcoplasmic reticulum calcium leak in ventricular myocytes isolated from patients with aortic stenosis and heart failure, providing the first cardiac surgery-relevant, human-tissue mechanistic evidence for GLP-1R-mediated electrophysiological cardioprotection [9]. Second, a 2026 observational study of patients undergoing isolated CABG reported a significant reduction in new-onset postoperative atrial fibrillation (POAF) among preoperative GLP-1 RA users, the first cardiac surgery-specific clinical outcome signal for a non-glycemic endpoint [10]. Third, the REVERSE-TAVR trial (NCT07090343) was formally registered in 2025 as the first randomized controlled trial specifically designed to evaluate semaglutide as a cardioprotective agent in a cardiac procedural population [11].

It is important to clarify what this review is and is not. The cardiovascular outcome trial literature on GLP-1 RAs is well-established and has been reviewed extensively elsewhere. We use it as mechanistic and contextual scaffolding to address a specifically underexplored question: what does the GLP-1 RA receptor biology, and the new surgical outcome and translational data of 2025–2026, collectively imply for how we should think about these agents in the perioperative management of cardiac surgery patients? This is a question that the ambulatory cardiovascular literature was neither designed nor positioned to answer.

This review is structured around four objectives: (1) to delineate the mechanistic basis for GLP-1 RA cardioprotection specifically relevant to the physiological challenges of cardiac surgery with cardiopulmonary bypass; (2) to critically appraise all existing clinical evidence in the perioperative cardiac surgery setting; (3) to evaluate evolving perioperative management guidance, including the updated multi-society approach to the aspiration controversy; and (4) to propose a tiered research agenda that maps the path from current observational signals to adequately powered randomized trials.

## 2. Review Methodology

This article is a narrative review and was not conducted as a systematic review or meta-analysis. We searched PubMed/MEDLINE, Embase, Scopus, the Cochrane Library, and ClinicalTrials.gov from database inception to 30 April 2026, combining the terms “GLP-1 receptor agonist”, “semaglutide”, “liraglutide”, “dulaglutide”, “exenatide”, “cardiac surgery”, “cardiopulmonary bypass”, “coronary artery bypass grafting”, “aortic valve replacement”, “TAVR”, “cardioprotection”, “ischemia–reperfusion”, “postoperative atrial fibrillation”, and “perioperative management”. We prioritized human mechanistic, translational, observational, and randomized studies of direct relevance to the cardiac surgical and perioperative setting, supplemented by seminal cardiovascular outcome trials and multi-society guidance for context, and we manually screened the reference lists of key articles. No formal risk-of-bias scoring or quantitative synthesis was undertaken; study selection was purposive and reflects the interpretive nature of a narrative review.

## 3. Biological Mechanisms of Cardioprotection

### 3.1. GLP-1 Receptor Distribution and Cardiac Signaling

**Figure 1:**
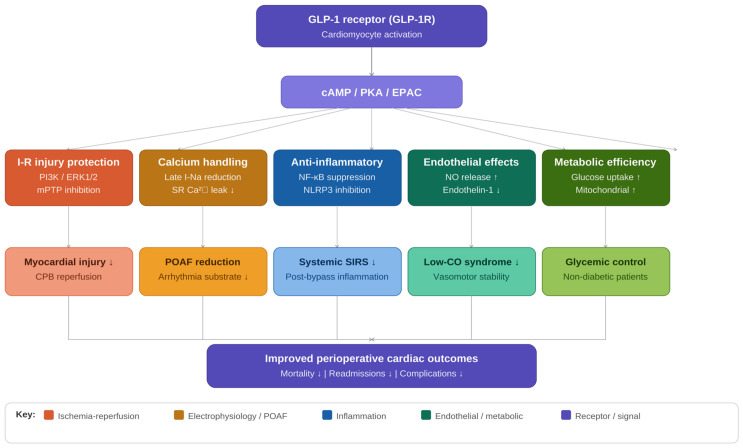
Mechanistic framework of GLP-1 receptor agonist cardioprotection in the cardiac surgical context. GLP-1 receptor (GLP-1R) activation in cardiomyocytes triggers adenylyl cyclase (AC)-mediated cyclic AMP (cAMP) elevation, activating protein kinase A (PKA) and exchange proteins directly activated by cAMP (EPAC). Downstream effects include: (1) phospholamban phosphorylation improving sarcoplasmic reticulum calcium re-uptake; (2) RISK pathway (PI3K/ERK1/2) activation inhibiting mitochondrial permeability transition pore (mPTP) opening during reperfusion; (3) late sodium current (I-Na-late) reduction limiting calcium overload and arrhythmia substrate; (4) NF-kB and NLRP3 inflammasome suppression attenuating post-bypass systemic inflammation; and (5) insulin-independent glucose uptake improving myocardial metabolic efficiency. All pathways depicted are derived from mechanistic, preclinical, or human-tissue electrophysiology studies and represent biological rationale; they have not been demonstrated as clinical effects in cardiac surgery populations and should be interpreted as hypothetical at the clinical level.

The GLP-1 receptor (GLP-1R) is a G-protein-coupled receptor expressed not only in pancreatic beta cells but also in cardiomyocytes, vascular smooth muscle cells, endothelial cells, and cardiac conduction tissue [12]. Ligand binding activates adenylyl cyclase, elevates cyclic AMP (cAMP), and subsequently engages protein kinase A (PKA) and exchange protein directly activated by cAMP (EPAC) pathways. In the cardiomyocyte, these cascades converge on improved calcium handling, mitochondrial biogenesis, and suppression of apoptotic signaling [13]. GLP-1R activation also increases myocardial glucose uptake through insulin-independent mechanisms, an effect particularly relevant in the energy-stressed perioperative myocardium [14] (Table 1, Figure 1).

### 3.2. Ischemia–Reperfusion Injury Attenuation

Ischemia–reperfusion (I-R) injury is central to the pathophysiology of perioperative myocardial damage in cardiac surgery with cardiopulmonary bypass. GLP-1 RAs attenuate I-R injury through multiple, partially redundant pathways [15]. PKA-mediated phosphorylation of phospholamban enhances sarcoplasmic reticulum calcium re-uptake, limiting cytosolic calcium overload during reperfusion. GLP-1R activation also activates the reperfusion injury salvage kinase (RISK) pathway, including phosphoinositide 3-kinase (PI3K) and extracellular signal-regulated kinase (ERK1/2), which collectively inhibit mitochondrial permeability transition pore (mPTP) opening [16]. In animal models, exendin-4 and liraglutide both reduced infarct size when administered prior to or during reperfusion [17]. Importantly, this protection is not glucose-dependent, meaning it extends to euglycemic non-diabetic patients undergoing cardiac surgery [14].

### 3.3. Anti-Inflammatory and Endothelial Effects

Cardiac surgery triggers a systemic inflammatory response characterized by complement activation, cytokine release, and neutrophil-mediated endothelial injury. GLP-1 RAs suppress NF-kB-driven inflammatory cascades, reduce circulating interleukin-6 (IL-6) and tumor necrosis factor-alpha (TNF-alpha), and attenuate the NLRP3 inflammasome, a key mediator of sterile inflammation in the post-bypass state [18,19]. In a pressure-overload cardiac hypertrophy model, semaglutide ameliorated pathological remodeling through NLRP3 inflammasome suppression via mitophagy enhancement [20]. Endothelial stabilization through GLP-1R-mediated nitric oxide (NO) release and reduced endothelin-1 production further attenuates the perioperative vasomotor dysfunction that contributes to low-cardiac-output syndrome [21].

### 3.4. Direct Electrophysiological Effects and the POAF Mechanism

The electrophysiological dimension of GLP-1R-mediated cardioprotection is the most directly relevant to cardiac surgery and the most mechanistically specific. In 2025, Krammer and colleagues published the first direct electrophysiological characterization of semaglutide in isolated human ventricular cardiomyocytes from patients with aortic stenosis and HFrEF [9]. Semaglutide significantly reduced late sodium current (I-Na-late) in both phenotypes. Late I-Na is a pathological current that accumulates during cellular stress, driving sodium-calcium exchanger-mediated calcium overload, SR calcium leak, triggered activity, and afterdepolarizations—the cellular substrate of POAF. Semaglutide also reduced SR calcium leak directly and improved contractility, effects mediated via the GLP-1R-cAMP-CaMKII signaling axis rather than through glycemic modulation. These effects occurred in euglycemic cardiomyocytes, confirming that the mechanism is glucose-independent and, therefore, applicable to the non-diabetic surgical patient. The clinical translation of these findings was confirmed in a 2026 observational CABG cohort, in which preoperative GLP-1 RA use was independently associated with a reduced incidence of new-onset POAF [10]. POAF complicates 20–40% of cardiac surgery cases, is a leading driver of stroke, prolonged hospitalization, and readmission, and currently lacks a pharmacological prevention strategy with proven perioperative efficacy [22]. The convergence of the Krammer mechanistic data and the 2026 CABG outcome signal constitutes the first human-tissue-to-clinical-outcome mechanistic chain for GLP-1 RA cardioprotection in a cardiac surgery-specific context.

## 4. Clinical Evidence in the Cardiac Surgery Setting

### 4.1. The GLOBE Randomized Trial

The GLOBE trial (NCT01190839) represents the most rigorously designed randomized controlled evidence for GLP-1 RA use in cardiac surgery to date [23] (Table 2). Patients undergoing elective cardiac surgery received two preoperative subcutaneous injections of liraglutide (0.6 mg the evening before surgery and 1.2 mg at induction) or matching placebo. Liraglutide significantly improved perioperative glycemic control without increasing hypoglycemia risk compared with conventional insulin protocols. A pre-planned secondary analysis evaluated the impact on myocardial function through postoperative echocardiography, hemodynamic parameters, and biomarkers of cardiac injury [24]. While liraglutide reduced peak postoperative glucose levels and improved intraoperative glycemic stability, significant improvements in echocardiographic parameters of systolic or diastolic function were not demonstrated at the timepoints assessed. Continuous glucose monitoring data from the GLOBE sub-study confirmed the superior glucose trajectory with liraglutide compared to placebo [25].

The GLOBE trial has critical methodological limitations when viewed through a cardioprotection lens. Liraglutide is a daily dosed formulation with a plasma half-life of approximately 13 h, which may be insufficient to achieve receptor saturation and full downstream cardioprotective signaling at the doses and timing employed. The trial was underpowered for hard cardiac endpoints and predates the semaglutide era. These limitations highlight the need for adequately powered, modern-agent trials. Critically, GLOBE was designed and powered for perioperative glycemic control as its primary endpoint and was not intended or statistically powered to detect hard cardioprotective outcomes. Its results should, therefore, be read as evidence of safe and effective perioperative glucose management, not as confirmation or refutation of a perioperative cardioprotective effect.

### 4.2. Observational CABG Data: The Postoperative Atrial Fibrillation Signal

A 2026 observational study from a prospectively maintained institutional cardiac surgery database examined outcomes in patients undergoing non-emergent isolated CABG from 2010 to 2024, stratified by preoperative GLP-1 RA use [10]. After multivariable Cox regression adjustment, GLP-1 RA use was not associated with differences in all-cause mortality or MACE, but was significantly associated with a lower risk of new-onset POAF. This finding is biologically plausible given the electrophysiological effects of GLP-1R activation described above, particularly the reduction in late I-Na and SR calcium leaks that are key substrates for the reentrant arrhythmia driving POAF. A parallel database study in adult congenital heart disease patients undergoing CABG using the TriNetX Research Network found a significant reduction in one-year hospital readmission rates with perioperative GLP-1 RA use, without increased complication risk [26]. These observational findings are hypothesis-generating and cannot establish a causal relationship. Both cohorts are susceptible to confounding by indication, immortal-time bias, selection bias, and exposure misclassification, and the pooling of pharmacokinetically distinct GLP-1 RAs introduces further heterogeneity. The reduction in POAF should, therefore, be regarded as an association requiring confirmation in adequately powered randomized trials rather than as evidence of a causal, agent-specific effect.

### 4.3. The REVERSE-TAVR Trial

The REVERSE-TAVR trial (NCT07090343; Semaglutide for Reducing Cardiovascular Events in Patients Undergoing Transcatheter Aortic Valve Replacement) is the first randomized trial specifically powered to evaluate a GLP-1 RA as a cardioprotective agent in a cardiac procedure population [11]. Led by Leiden University Medical Center and scheduled to begin enrollment in April 2026, REVERSE-TAVR uses semaglutide in a TAVR population with cardiovascular events as the primary endpoint. The biological rationale is well-supported: human cardiomyocyte data from patients with aortic stenosis demonstrate that semaglutide reduces late I-Na and SR calcium leak in the disease-specific cellular milieu [9]. Results expected by 2030 will determine whether GLP-1 RAs reduce cardiovascular events in this high-risk procedural population.

### 4.4. Evidence in Non-Cardiac Surgery and Translational Data

A systematic review and meta-analysis published in eClinicalMedicine (2025) evaluated preoperative GLP-1 RA safety across elective surgical procedures, concluding that perioperative GLP-1 RA use was not associated with increased complications when current multisociety guidance on gastric emptying was followed [27]. A Columbia University cohort study of 392,065 patients demonstrated no association between GLP-1 RA use and pulmonary aspiration risk after risk-adjusted analysis [28]. A large observational study using the IBM MarketScan database, including 39,516 CABG patients, reported that preoperative GLP-1 RA exposure was associated with reduced postoperative ileus [29]. An ongoing multicenter RCT from the University of Hong Kong (NCT06324461) is evaluating subcutaneous dulaglutide for reduction in myocardial injury after non-cardiac surgery [30].

## 5. Perioperative Management Considerations

### 5.1. Perioperative Safety: From Blanket Cessation to Individualized Risk Assessment

The perioperative safety of GLP-1 RAs has undergone substantial re-evaluation since 2024, and recent evidence does not support routine preoperative discontinuation in all patients, although an individualized aspiration-risk assessment remains necessary. The original rationale for blanket preoperative GLP-1 RA cessation rested on the observation that these agents delay gastric emptying through vagal afferent signaling [31]. However, this theoretical concern has not been borne out at the clinical level. A large observational study of 392,065 surgical patients found no association between GLP-1 RA use and pulmonary aspiration after risk adjustment (OR 1.07; 95% CI 0.85–1.34) [28]. A parallel systematic review and meta-analysis of perioperative GLP-1 RA safety across 246,242 patients confirmed no increase in overall perioperative complication rates when current guidance was followed [27]. In 2025, a multi-society consensus statement formally replaced the blanket preoperative cessation recommendation with an individualized, shared decision-making framework [32]. From a cardiac surgery-specific standpoint, the relevant remaining safety considerations are not aspiration per se but rather the pharmacokinetic optimization of preoperative timing, the interaction between GLP-1 RA-mediated natriuresis and intraoperative fluid management, and the need for protocolized metabolic monitoring in the immediate post-CPB period. Aspiration precautions nonetheless remain warranted in selected higher-risk patients, including those with active gastrointestinal symptoms (nausea, vomiting, or early satiety), recent dose escalation, high-dose therapy, severe obesity, diabetic gastroparesis, or non-elective, non-fasted (emergency) surgery, in whom a longer preoperative hold, point-of-care gastric ultrasound, or modified airway management may be appropriate. The shift is, therefore, from blanket cessation toward individualized, risk-stratified management rather than an abolition of aspiration risk.

### 5.2. Drug-Specific Pharmacokinetic Considerations

The heterogeneity of GLP-1 RA pharmacokinetics has critical perioperative implications (Table 3). Daily dosed agents (liraglutide, exenatide) reach steady-state more rapidly and clear more quickly, making them more amenable to standard preoperative withdrawal windows [7]. Weekly dosed agents (semaglutide, dulaglutide) have half-lives of approximately 5–7 days, meaning that a standard one-week preoperative hold does not guarantee full gastric normalization. Conversely, from a cardioprotective standpoint, the prolonged receptor occupancy of weekly semaglutide may confer superior and more sustained preconditioning of the surgical myocardium compared with short-acting alternatives [2,9]. The timing, duration, and agent choice for perioperative GLP-1 RA use thus represents a pharmacological optimization problem that current guidelines do not yet address in the cardiac surgery-specific context.

Recommendations synthesize current multi-society guidance and pharmacokinetic principles; they are not validated cardiac surgery-specific protocols and must be individualized to the patient and procedure. In non-diabetic patients receiving GLP-1 RAs solely for cardiometabolic indications, the timing of postoperative reinitiation is less time-critical. GI, gastrointestinal; IR, immediate-release; ER, extended-release [7,32].

### 5.3. The Non-Diabetic Cardiac Surgery Patient: An Unexplored Frontier

The non-diabetic cardiac surgery patient represents arguably the most underdeveloped and clinically important frontier in the GLP-1 RA perioperative literature—and one that is absent from virtually all existing reviews. This population deserves dedicated attention for three reasons. First, it is numerically dominant: the majority of patients undergoing complex aortic surgery, isolated CABG in patients with preserved metabolic function, and reoperative cardiac procedures are not diabetic. Second, they experience the full physiological burden of cardiac surgery with CPB (I-R injury, systemic inflammation, cardioplegia-related metabolic stress) yet derive no glycemic benefit from GLP-1 RA therapy and would, therefore, receive it purely as a cardioprotective preconditioning agent. Third, as demonstrated in the Krammer 2025 cardiomyocyte data, GLP-1R-mediated electrophysiological protection is glucose-independent and operates through a cAMP-CaMKII mechanism that does not require hyperglycemia as a substrate [9]. The GLOBE trial established glycemic benefit in mixed surgical populations, and secondary analyses confirmed cardioprotective signals independent of baseline glycemic status [23,24]. The 2024 ESC guidelines for chronic coronary syndrome now formally recommend semaglutide in overweight or obese patients without diabetes for cardiovascular risk reduction, establishing a non-glycemic indication precedent [5]. We argue that the non-diabetic complex cardiac surgery patient should be a primary, not secondary, target population for future GLP-1 RA perioperative investigation. It must be emphasized, however, that the rationale for prioritizing non-diabetic patients is at present indirect, extrapolated largely from cardiovascular outcome trials and mechanistic studies, and that a perioperative cardioprotective benefit has not been established in this population. This argument is, therefore, advanced as a hypothesis to be tested in dedicated studies rather than as a demonstrated effect.

### 5.4. Effects Beyond the Heart: Multi-Organ Considerations

Beyond the myocardium, GLP-1 RAs exert effects across multiple organ systems that are relevant to perioperative risk assessment. Renal protection is increasingly well established: in a dedicated kidney-outcome trial, semaglutide reduced the progression of chronic kidney disease and major kidney events, an effect of particular interest given the high incidence of cardiac surgery-associated acute kidney injury [33]. Ophthalmologic safety warrants attention, because rapid glycemic improvement has been associated with early worsening of diabetic retinopathy in some trials, a consideration mainly in diabetic patients with pre-existing retinopathy [34]. Gastrointestinal and pancreaticobiliary effects include nausea, delayed gastric emptying, an increased incidence of cholelithiasis and biliary events, and rare reports of acute pancreatitis [35]. A labeled precaution exists regarding medullary thyroid C-cell tumors, derived from a rodent finding of GLP-1 receptor-mediated C-cell activation and calcitonin release; however, human thyroid C-cells express the receptor at very low levels and this signal has not been confirmed in humans [36]. Hepatic effects are generally favorable, with improvement in metabolic dysfunction-associated steatotic liver disease [37]. Pulmonary considerations in the surgical setting relate primarily to aspiration risk, addressed above, rather than to direct pulmonary toxicity. These multi-organ effects are largely favorable or neutral but should inform individualized perioperative decision-making.

### 5.5. Body Composition, Lean-Mass Loss, and the Older Surgical Patient

GLP-1 RA-induced weight loss is substantial and includes a meaningful loss of lean body mass, with body-composition sub-studies indicating that a significant proportion of the total weight lost is fat-free mass [38]. In the cardiac surgical population, where sarcopenia and frailty are already strong predictors of mortality, prolonged ventilation, and failure to rescue, accelerated lean-mass and skeletal-muscle loss is a potentially important and underrecognized concern. Older patients with pre-existing malnutrition or low muscle reserve are most vulnerable, particularly when the postoperative course is complicated by prolonged immobilization, intensive care stay, or catabolic stress, which can compound muscle wasting and impair rehabilitation. Conversely, in obese patients, preoperative weight reduction may improve operative exposure, reduce sternal wound complications, and lower the technical difficulty of surgery. We, therefore, suggest pairing preoperative GLP-1 RA use with nutritional optimization, protein supplementation, and resistance-oriented prehabilitation where feasible, and formally assessing frailty and nutritional status before initiating or continuing these agents in older surgical candidates.

### 5.6. Effect Modifiers: Sex, Body Mass Index, and Age

The magnitude of GLP-1 RA effects may vary by sex, body mass index, and age, and these modifiers have not been examined in cardiac surgical cohorts. Women generally experience greater weight loss and a higher incidence of gastrointestinal adverse effects than men at equivalent doses, which may influence both aspiration risk and tolerability. Higher baseline body mass index is associated with larger absolute weight reduction but also with a greater burden of obesity-related operative risk, and the net perioperative effect of these opposing influences is unknown. Older patients may be more susceptible to volume depletion, lean-mass loss, and the hemodynamic consequences of reduced oral intake. Future observational and randomized work should pre-specify analyses stratified by sex, body mass index, and age to identify the subgroups in whom the perioperative risk-benefit balance is most favorable.

### 5.7. Perioperative Drug–Drug Interactions

GLP-1 RAs have few pharmacokinetic interactions mediated by hepatic cytochrome enzymes, but their effect on gastric emptying can alter the rate, and occasionally the extent, of absorption of co-administered oral medications, including antihypertensives, thyroid hormone, and oral anticoagulants, which becomes relevant when the oral regimen is reintroduced postoperatively [2,7]. Pharmacodynamic interactions are more clinically important. Co-administration with insulin or sulfonylureas increases the risk of perioperative hypoglycemia and warrants dose reduction in background glucose-lowering therapy [7]. Combination with sodium-glucose cotransporter-2 inhibitors raises the risk of euglycemic diabetic ketoacidosis and additive volume depletion, both of which intersect with perioperative fasting and fluid shifts [7]. Beta-blockers may blunt the modest chronotropic effect of GLP-1 RAs, whereas angiotensin receptor-neprilysin inhibitors and other vasodilators may exert additive hypotensive and natriuretic effects relevant to intraoperative and early postoperative hemodynamic management. Thyroid hormone replacement should be monitored, given both altered absorption and the labeled thyroid precautions noted above.

### 5.8. Side-Effect Burden and Surgery-Specific Risks

The characteristic gastrointestinal side effects of GLP-1 RAs, including nausea, vomiting, and reduced oral intake, can contribute to perioperative dehydration, electrolyte disturbance, and delayed return to adequate nutrition, all of which are relevant to the recovering cardiac surgical patient. Notably, observational data suggest a reduction rather than an increase in postoperative ileus with preoperative GLP-1 RA exposure, possibly reflecting improved glycemic and metabolic control [29]. Evidence on wound healing is limited and largely preclinical; experimental models, predominantly of diabetic wounds, suggest neutral-to-favorable effects mediated by improved glycemic control and anti-inflammatory signaling, but direct data on sternal or vascular surgical wound healing are lacking. Similarly, a potential platelet-modulating or antiplatelet effect of GLP-1 RAs has been described in preclinical and small mechanistic studies, but its clinical significance for postoperative bleeding, transfusion, or re-exploration after cardiac surgery is unknown and has not been demonstrated in surgical cohorts [39,40,41]. These surgery-specific effects should be regarded as hypothesis-generating and represent priorities for dedicated investigation rather than established risks or benefits.

### 5.9. Chronotropic and Arrhythmic Effects in the Postoperative Setting

GLP-1 RAs produce a modest increase in resting heart rate, typically in the order of a few beats per minute, and have been associated with a small increase in atrial and ventricular ectopy in some studies [34]. In the immediate postoperative period, when patients are exposed to volume shifts, electrolyte derangements (notably hypokalemia and hypomagnesemia), catecholamine stress, and an arrhythmogenic substrate, this chronotropic effect could theoretically interact unfavorably, although it must be weighed against the electrophysiological and POAF-protective mechanisms discussed earlier. The net arrhythmic effect of GLP-1 RAs in the perioperative cardiac surgical setting is, therefore, uncertain and potentially bidirectional, reinforcing the need for telemetry, attentive electrolyte repletion, and prospective arrhythmia-focused endpoints in future trials.

### 5.10. The HFrEF Patient: A Cautionary Signal

Although the mechanistic data discussed above include favorable electrophysiological effects of semaglutide in cardiomyocytes from patients with heart failure with reduced ejection fraction (HFrEF), the clinical trial evidence in advanced HFrEF is more cautionary and must be reconciled with that mechanistic optimism. In randomized trials of liraglutide in advanced or recently decompensated HFrEF, GLP-1 RA therapy did not improve clinical stability and was accompanied by an increase in heart rate and numerically higher rates of serious cardiac adverse events, including arrhythmic and worsening-heart-failure events [42,43]. The mechanisms underlying this apparent discrepancy between isolated-myocyte benefit and an adverse clinical signal in advanced HFrEF are incompletely understood and may relate to chronotropic effects, increased myocardial energy demand, or volume changes in a vulnerable population. For the cardiac surgical patient with advanced or decompensated HFrEF, these data warrant particular caution, and GLP-1 RAs should not be assumed to be cardioprotective in this subgroup on the basis of mechanistic data alone. This tension is itself an argument for dedicated, ejection-fraction-stratified prospective study.

### 5.11. Cost, Equity, and Ethical Considerations

The high acquisition cost of branded GLP-1 RAs and their variable insurance coverage raise equity and access concerns relevant to any proposal to use these agents for perioperative cardioprotection. If a perioperative cardioprotective benefit were established, restricting access on the basis of cost could widen existing disparities in cardiac surgical outcomes, given that patients of lower socioeconomic status already experience worse perioperative outcomes [44]. Ongoing supply constraints, off-label and compounded use, and the prioritization of these agents for weight management over cardiometabolic indications further complicate equitable allocation. Any future perioperative protocol should, therefore, be accompanied by explicit consideration of cost-effectiveness and equitable access, and trials should report outcomes across socioeconomic and demographic strata. The ethical introduction of GLP-1 RAs as perioperative cardioprotective agents will depend not only on demonstrating efficacy but on ensuring that benefit is distributed fairly.

## 6. Evidence Gaps and Research Agenda

The evidence base for GLP-1 RAs in cardiac surgery is characterized by three distinct asymmetries: a strong mechanistic foundation, an early and directionally consistent clinical signal, and a near-complete absence of prospective interventional data. Resolving this asymmetry requires a structured, staged research agenda.

First, and most urgently, the field needs adequately powered randomized trials using contemporary weekly dosed agents with cardiac surgery-specific cardioprotection endpoints. All existing surgical outcome data are observational. The GLOBE trial used liraglutide at suboptimal doses with glycemic control as the primary endpoint, a design not built to detect cardioprotective effects. The REVERSE-TAVR trial (NCT07090343), scheduled to begin enrollment in April 2026, is the first RCT designed to address this gap [11]. REVERSE-TAVR is the benchmark, but open cardiac surgery, including CABG, valve replacement, and complex aortic surgery, requires its own dedicated RCT design. Future open cardiac surgery trials should adopt troponin area under the curve (AUC) as the primary endpoint, with POAF incidence, ICU length of stay, and 30-day MACE as co-primary or key secondary endpoints.

Second, no outcome data exist in open cardiac surgery populations with high I-R injury burden: complex aortic procedures requiring deep hypothermic circulatory arrest, preoperative CABG, and combined valve-CABG cases. These are precisely the operations where GLP-1 RA cardioprotection would theoretically be most impactful, given the severity of the ischemic insult, the duration of CPB, and the magnitude of the post-bypass inflammatory response. Open aortic surgery should be an explicit target population for phase II proof-of-concept RCTs.

Third, the non-diabetic cardiac surgery patient must be a primary focus of future investigation. No existing trial, guideline, or systematic review addresses this group specifically. Dedicated non-diabetic cohort analyses within large database studies (STS National Database, Epic Cosmos) should be conducted before RCT design to establish baseline event rates and identify patient subgroups with the greatest potential for benefit.

Fourth, the optimal perioperative pharmacological strategy (agent, dose, initiation timing, and duration) has not been defined for any cardiac surgery population. Pharmacokinetic modeling studies, followed by dose-ranging phase II RCTs, are needed before phase III designs can be adequately powered.

**Figure 2:**
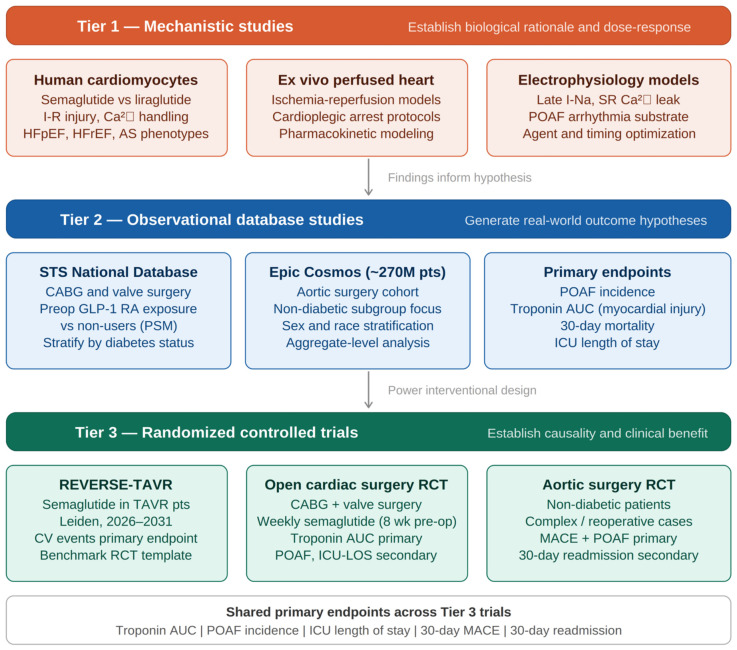
Proposed three-tiered research agenda for GLP-1 receptor agonists as perioperative cardioprotective agents in cardiac surgery. Tier 1: Mechanistic studies; human cardiomyocyte and ex vivo perfused heart models comparing semaglutide vs. liraglutide on I-R injury biomarkers and calcium handling across surgical phenotypes (HFpEF, HFrEF, aortic stenosis). Tier 2: Observational database analyses using the STS National Database and Epic Cosmos (~270 million patients) linking preoperative GLP-1 RA exposure to POAF incidence, troponin AUC, and 30-day outcomes across CABG, valve replacement, and open aortic surgery cohorts stratified by diabetic status. Tier 3: Randomized controlled trials beginning with the REVERSE-TAVR benchmark and extending to open cardiac surgery and aortic surgery populations, with troponin AUC and POAF as co-primary endpoints. In this framework, Tier 1 reflects mechanistic and translational evidence, Tier 2 reflects observational data, and Tier 3 reflects hypothetical, not-yet-conducted trials. The figure is a proposed research agenda and does not depict established clinical practice or proven benefit.

We propose a three-tiered research agenda (Figure 2) to address these gaps in sequence. Tier 1 consists of mechanistic and translational studies: ex vivo perfused heart models using semaglutide vs. liraglutide in CPB-simulating conditions, stratified by diabetic status and surgical phenotype. Tier 2 consists of large database observational analyses using the STS National Database and Epic Cosmos (~270 million patients), linking preoperative GLP-1 RA exposure to POAF incidence, troponin AUC, 30-day mortality, and readmission rates, with mandatory diabetic/non-diabetic stratification [45]. Tier 3 consists of adequately powered RCTs, beginning with REVERSE-TAVR and extending to open cardiac surgery and aortic surgery-specific trials, with mandatory enrollment of non-diabetic patients constituting at least 40% of the study population.

## 7. Discussion

This narrative review synthesizes a growing body of mechanistic, translational, and emerging clinical evidence to argue that GLP-1 RAs warrant reframing from purely metabolic drugs to potential perioperative cardioprotective agents in cardiac surgery. The biological rationale is robust: GLP-1R activation converges on multiple pathways directly relevant to the physiological challenges of cardiac surgery with cardiopulmonary bypass, including I-R injury, systemic inflammation, calcium overload, and arrhythmia substrate. The electrophysiological effects of semaglutide on late I-Na and SR calcium handling, demonstrated directly in human cardiomyocytes from aortic stenosis and HFrEF patients, are particularly compelling given the mechanistic link to POAF [9].

The clinical evidence, while early, is directionally consistent. The GLOBE trial established perioperative glycemic benefit and safety, the CABG observational data identified a POAF reduction signal, the CHD-CABG database study showed readmission benefit, and REVERSE-TAVR represents the field’s first dedicated RCT. Importantly, the safety profile of GLP-1 RAs in the perioperative setting has become better defined: updated multi-society guidance has moved away from blanket preoperative cessation toward individualized risk assessment, and large observational data have not confirmed the aspiration risk that initially drove blanket cessation policies [27,28,32].

The concept of pharmacological cardioprotection in cardiac surgery is not new: remote ischemic preconditioning, volatile anesthetic preconditioning, and tight glycemic control have all been explored with varying degrees of success [46]. What distinguishes the GLP-1 RA story is the breadth of the mechanistic rationale, the drug class’s proven cardiovascular benefit in non-surgical populations, and the fact that many patients undergoing cardiac surgery are already taking these agents or represent candidates for them on independent cardiometabolic grounds. This convergence of existing prescription, proven cardiac benefit, and mechanistic cardioprotective plausibility is unusual in the history of perioperative cardioprotective drug development.

A note of appropriate caution is warranted. The GLOBE trial did not demonstrate significant improvement in cardiac function endpoints, and observational CABG data are susceptible to confounding by indication. The REVERSE-TAVR trial is critical to establishing causality. Until those data emerge, the evidence reviewed here should be interpreted as hypothesis-generating and sufficient to justify further investigation, not as definitive proof of clinical benefit in cardiac surgery populations. Accordingly, the present synthesis is intended to motivate and structure future research, not to change current clinical practice; a definitive perioperative cardioprotective benefit of GLP-1 RAs in cardiac surgery has not been established, and routine perioperative use of these agents for cardioprotection cannot be recommended on the basis of the evidence reviewed here.

## 8. Conclusions

GLP-1 receptor agonists are no longer merely glucose-lowering drugs. Their receptor-mediated cardioprotective biology, their proven cardiovascular benefit across a spectrum of cardiometabolic phenotypes, and their early perioperative cardiac surgery signals collectively make a compelling case for systematic investigation in the cardiac surgical setting. The reduction in POAF observed in CABG patients, the direct human cardiomyocyte data, and the REVERSE-TAVR trial design all point toward a paradigm in which GLP-1 RAs are considered not only for metabolic optimization but as pharmacological tools to reduce perioperative myocardial injury, arrhythmia burden, and cardiovascular morbidity. Critical evidence gaps remain, particularly for open cardiac surgery, aortic surgery, and non-diabetic populations. We propose a structured three-tiered research agenda beginning with large observational database analyses and culminating in adequately powered randomized trials using contemporary weekly dosed agents. The cardiac surgery community is at an inflection point: the biological and early clinical evidence has accumulated to the point where systematic, adequately powered prospective investigation is clearly warranted. The field is ready for the next generation of perioperative cardioprotection trials, and GLP-1 receptor agonists deserve a central place in that agenda.

## Figures and Tables

**Table 1:** Key cardioprotective mechanisms of GLP-1 receptor agonists and their perioperative relevance in cardiac surgery.

Mechanism	Molecular Pathway	Perioperative Relevance
Ischemia–reperfusion protection	PI3K/ERK1/2 (RISK pathway); mPTP inhibition; PKA-phospholamban axis	Cardioplegia arrest and reperfusion during CPB; myocardial stunning
Anti-inflammatory signaling	NF-kB suppression; IL-6, TNF-alpha reduction; NLRP3 inflammasome inhibition	Post-bypass systemic inflammatory response syndrome (SIRS)
Calcium and sodium handling	Reduction in late I-Na; SR calcium leak; GLP-1R-cAMP-CaMKII axis	POAF prevention; diastolic dysfunction in aortic stenosis and HFpEF
Endothelial stabilization	NO release; endothelin-1 reduction; endothelial-derived hyperpolarizing factor	Vasomotor dysfunction; low-cardiac-output syndrome
Myocardial metabolic efficiency	GLP-1R-mediated glucose uptake (insulin-independent); mitochondrial biogenesis	Energy-starved perioperative myocardium; non-diabetic patients on CPB
Natriuretic and renal effects	Proximal tubule sodium reabsorption inhibition; volume regulation	Perioperative fluid overload; pulmonary edema post-CPB

CPB, cardiopulmonary bypass; POAF, postoperative atrial fibrillation; HFpEF, heart failure with preserved ejection fraction; SR, sarcoplasmic reticulum; mPTP, mitochondrial permeability transition pore; NO, nitric oxide.

**Table 2:** Summary of key clinical evidence on GLP-1 receptor agonists in the cardiac surgery and perioperative setting.

Study	Design	Population	Agent	Key Finding	Limitation
GLOBE Trial [23]	RCT	Elective cardiac surgery	Liraglutide (daily)	Improved perioperative glycemic control; no hypoglycemia increase	Underpowered for cardiac endpoints; short-acting agent; pre-semaglutide era|Primary endpoint glycemic (not cardioprotective); exposure = two fixed preoperative liraglutide doses; randomized but not propensity-matched; n = 278 randomized
GLOBE Cardiac Function Sub-study [24]	RCT secondary analysis	Elective cardiac surgery	Liraglutide (daily)	No significant improvement in echocardiographic cardiac function|Pre-planned secondary analysis; surrogate echocardiographic/functional endpoints; not powered for these endpoints; n = 261 (intention-to-treat); echocardiography in 170	Functional endpoints; timing and dose of liraglutide may be suboptimal
GLP-1 and POAF in CABG [10]	Observational cohort	Isolated CABG (2010–2024)	Multiple GLP-1 RAs	Significant reduction in new-onset POAF; no mortality difference	Retrospective; confounding by indication; limited by GLP-1 RA heterogeneity|Single-institution; exposure = any preoperative GLP-1 RA prescription (agent-heterogeneous, possible misclassification); multivariable Cox adjustment but susceptible to immortal-time bias; not propensity-matched; n = 4170 (165 GLP-1 users)
CABG readmission in CHD [26]	Retrospective cohort (TriNetX)	Adult CHD undergoing CABG	Multiple GLP-1 RAs	Significant reduction in 1-year readmission; no increased complications	Administrative database; CHD-specific population limits generalizability|Claims-based exposure ascertainment; readmission is an administrative endpoint; propensity-matched within network but residual confounding likely; 1:1 propensity-matched TriNetX cohorts
REVERSE-TAVR [11]	RCT (planned 2026–2031)	TAVR patients	Semaglutide (weekly)	Primary endpoint: cardiovascular events post-TAVR (results pending)	Not yet enrolling; TAVR-specific; open surgery populations excluded|Planned RCT; cardiovascular-event primary endpoint; randomized exposure to weekly semaglutide; results pending; target enrollment per registry (NCT07090343)
Perioperative safety meta-analysis [27]	Systematic review and meta-analysis	Elective surgery (all types)	Multiple GLP-1 RAs	No increased perioperative complication rate with GLP-1 RA use	Heterogeneous surgical populations; cardiac surgery subgroup underpowered|246,242 patients pooled; safety endpoints (aspiration/complications), not efficacy; exposure definitions varied across studies; mixed adjustment methods

RCT, randomized controlled trial; CABG, coronary artery bypass grafting; CHD, congenital heart disease; POAF, postoperative atrial fibrillation; TAVR, transcatheter aortic valve replacement; CV, cardiovascular; MACE, major adverse cardiovascular events.

**Table 3:** Agent-specific framework for perioperative management of GLP-1 receptor agonists, integrating pharmacokinetics with current individualized (non-blanket) guidance.

Agent (Formulation)	Approx. Half-Life	Suggested Preoperative Management (Standard Risk)	Higher-Risk Modification	Postoperative Reinitiation
Semaglutide, subcutaneous (weekly)	~5–7 days	Hold the weekly dose during the week of surgery (about 1 week)	Consider a longer hold and gastric assessment if GI symptoms, recent dose escalation, or high dose	Resume once GI function has recovered and oral intake is tolerated
Semaglutide, oral (daily)	~5–7 days	Hold on the day of surgery; the long half-life confers an extended functional washout	As above	Resume with oral intake
Tirzepatide (weekly, GIP/GLP-1)	~5 days	Hold the weekly dose during the week of surgery (about 1 week)	As above	Resume with oral intake
Dulaglutide (weekly)	~5 days	Hold the weekly dose during the week of surgery (about 1 week)	As above	Resume with oral intake
Liraglutide (daily)	~13 h	Hold on the day of surgery	Consider also holding the preceding evening dose in higher-risk patients	Resume with oral intake
Exenatide (immediate- or extended-release)	IR ~2.4 h; ER long	Hold immediate-release on the day of surgery; hold extended-release about 1–3 weeks	As above	Resume with oral intake

## Data Availability

No new data were created in this study.

## Abbreviations

CABG	Coronary artery bypass grafting
cAMP	Cyclic adenosine monophosphate
CPB	Cardiopulmonary bypass
EPAC	Exchange protein directly activated by cAMP
ERK1/2	Extracellular signal-regulated kinase 1/2
GLP-1	Glucagon-like peptide-1
GLP-1R	Glucagon-like peptide-1 receptor
GLP-1 RA	Glucagon-like peptide-1 receptor agonist
HFpEF	Heart failure with preserved ejection fraction
HFrEF	Heart failure with reduced ejection fraction
I-R	Ischemia–reperfusion
IL-6	Interleukin-6
MACE	Major adverse cardiovascular events
mPTP	Mitochondrial permeability transition pore
NF-kB	Nuclear factor kappa-light-chain-enhancer of activated B cells
NLRP3	NLR family pyrin domain containing 3
NO	Nitric oxide
PI3K	Phosphoinositide 3-kinase
PKA	Protein kinase A
POAF	Postoperative atrial fibrillation
RCT	Randomized controlled trial
RISK	Reperfusion injury salvage kinase
SR	Sarcoplasmic reticulum
STS	Society of Thoracic Surgeons
T2DM	Type 2 diabetes mellitus
TAVR	Transcatheter aortic valve replacement
TNF-alpha	Tumor necrosis factor-alpha

## References

[1] Nauck M.A., Meier J.J. (2018). Incretin hormones: Their role in health and disease. Diabetes Obes. Metab..

[2] Drucker D.J. (2006). The biology of incretin hormones. Cell Metab..

[3] Lincoff A.M., Brown-Frandsen K., Colhoun H.M., Deanfield J., Emerson S.S., Esbjerg S., Hardt-Lindberg S., Hovingh G.K., Kahn S.E., Kushner R.F. (2023). Semaglutide and cardiovascular outcomes in obesity without diabetes. N. Engl. J. Med..

[4] Marx N., Federici M., Schutt K., Müller-Wieland D., Di Angelantonio E., Herrington W.G., Ajjan R.A., Kautzky-Willer A., Rocca B., Sattar N. (2023). 2023 ESC Guidelines for the management of cardiovascular disease in patients with diabetes. Eur. Heart J..

[5] Kala P., Pudil R., Varvařovský I., Skalická H., Vrints C., Andreotti F., Koskinas K.C., Rossello X., Adamo M., Ainslie J. (2024). 2024 ESC Guidelines for the management of chronic coronary syndromes. Eur. Heart J..

[6] Shroyer A.L., Grover F.L., Hattler B., Collins J.F., McDonald G.O., Kozora E., Lucke J.C., Baltz J.H., Novitzky D. (2009). On-pump versus off-pump coronary-artery bypass surgery. N. Engl. J. Med..

[7] Goldenberg R.M., Gilbert J.D., Houlden R.L., Khan T.S., Makhija S., Mazer C.D., Trinacty J., Verma S. (2025). Perioperative and periprocedural management of GLP-1 receptor-based agonists and SGLT2 inhibitors. Curr. Med. Res. Opin..

[8] Wookey O., Galligan A., Wilkie B., MacIsaac A., Paratz E. (2025). Perioperative Use of GLP-1 Receptor Agonists in Patients Undergoing Cardiac Procedures: A Scoping Review. Heart Lung Circ..

[9] Krammer T., Baier M.J., Hegner P., Zschiedrich T., Lukas D., Wolf M., Le Phu C., Lutz V., Evert K., Kozakov K. (2025). Cardioprotective effects of semaglutide on isolated human ventricular myocardium. Eur. J. Heart Fail..

[10] Hasan I., Bessette L.G., Jacquemyn X., Wang Y., Subramaniam K., Hasan Z., Thoma F., Ogami T., Bonatti J., Kaczorowski D. (2026). Association of GLP-1 Inhibitors with Cardiovascular Outcomes in Patients Undergoing Coronary Artery Bypass Surgery. J. Thorac. Cardiovasc. Surg..

[11] Leiden University Medical Center (2025). Semaglutide for Reducing Cardiovascular Events in Patients Undergoing Transcatheter Aortic Valve Replacement (REVERSE-TAVR).

[12] Drucker D.J., Nauck M.A. (2006). The incretin system: Glucagon-like peptide-1 receptor agonists and dipeptidyl peptidase-4 inhibitors in type 2 diabetes. Lancet.

[13] Baggio L.L., Drucker D.J. (2007). Biology of incretins: GLP-1 and GIP. Gastroenterology.

[14] Ravassa S., Zudaire A., Diez J. (2012). GLP-1 and cardioprotection: From bench to bedside. Cardiovasc. Res..

[15] Giblett J.P., Axell R.G., White P.A., Clarke S.J., McCormick L., Read P.A., Reinhold J., Brown A.J., O’Sullivan M., West N.E.J. (2016). Glucagon-like peptide-1 derived cardioprotection does not utilize a KATP-channel dependent pathway. Cardiovasc. Diabetol..

[16] Lecour S. (2009). Activation of the protective survivor activating factor enhancement (SAFE) pathway against reperfusion injury. J. Mol. Cell. Cardiol..

[17] Sonne D.P., Engstrom T., Treiman M. (2008). Protective effects of GLP-1 analogues exendin-4 and GLP-1(9-36) amide against ischemia-reperfusion injury in rat heart. Regul. Pept..

[18] Mehdi S.F., Pusapati S., Anwar M.S., Lohana D., Kumar P., Nandula S.A., Nawaz F.K., Tracey K., Yang H., LeRoith D. (2023). Glucagon-like peptide-1: A multi-faceted anti-inflammatory agent. Front. Immunol..

[19] Rakipovski G., Rolin B., Nohr J., Klewe I., Frederiksen K.S., Augustin R., Hecksher-Sørensen J., Ingvorsen C., Polex-Wolf J., Knudsen L.B. (2018). The GLP-1 analogs liraglutide and semaglutide reduce atherosclerosis in ApoE^−/−^ and LDLR^−/−^ mice by a mechanism that includes inflammatory pathways. JACC Basic. Transl. Sci..

[20] He W., Wei J., Liu X., Zhang Z., Huang R., Jiang Z. (2024). Semaglutide ameliorates pressure overload-induced cardiac hypertrophy by improving cardiac mitophagy to suppress the activation of NLRP3 inflammasome. Sci. Rep..

[21] Erdogdu O., Nathanson D., Sjoholm A., Nyström T., Zhang Q. (2010). Exendin-4 stimulates proliferation of human coronary artery endothelial cells through eNOS-, PKA- and PI3K/Akt-dependent pathways and requires GLP-1 receptor. Mol. Cell. Endocrinol..

[22] Suero O.R., Ali A.K., Barron L.R., Segar M.W., Moon M.R., Chatterjee S. (2024). Postoperative atrial fibrillation (POAF) after cardiac surgery: Clinical practice review. J. Thorac. Dis..

[23] Hulst A.H., Visscher M.J., Godfried M.B., Thiel B., Gerritse B.M., Scohy T.V., Bouwman R.A., Willemsen M.G.A., Hollmann M.W., Preckel B. (2020). Liraglutide for perioperative management of hyperglycaemia in cardiac surgery patients: A multicentre randomized superiority trial. Diabetes Obes. Metab..

[24] Hulst A.H., Visscher M.J., Cherpanath T.G.V., van de Wouw L., Godfried M.B., Thiel B., Gerritse B.M., Scohy T.V., Bouwman R.A., Willemsen M.G.A. (2020). Effects of liraglutide on myocardial function after cardiac surgery: A secondary analysis of the randomised controlled GLOBE trial. Nutrients.

[25] Oosterom-Eijmael M.J.P., Hermanides J., van Raalte D.H., Kouw I.W.K., DeVries J.H., Hulst A.H. (2024). Continuous Glucose Monitoring and the Effect of Liraglutide in Cardiac Surgery Patients: A Substudy of the Randomized Controlled GLOBE Trial. J. Cardiothorac. Vasc. Anesth..

[26] Yan J.H., McKinnerney J., Al Janabi T., Chahal A., Malik A., Vranian M.N., Kashyap R. (2025). Perioperative use of GLP-1 receptor agonists lower readmission rates after coronary artery bypass grafting in adults with congenital heart disease. Cureus.

[27] Kamarajah S.K., Gudiozzi N., Findlay J.M., Lee M.J., Pinkney T., Markar S.R. (2025). Evaluation of safety of preoperative GLP-1 receptor agonists in patients undergoing elective surgery: A systematic review, meta-analysis and meta-regression. eClinicalMedicine.

[28] Wright J.D., Chen L., Xu X., Hur C., Matsuo K., Elkin E.B., Hershman D.L. (2025). Glucagon-like-peptide-1 (GLP-1) receptor agonist use and the risk of pulmonary aspiration in patients undergoing surgery. Int. J. Surg..

[29] Rashid Z., Woldesenbet S., Khalil M., Altaf A., Kawashima J., Mumtaz K., Pawlik T.M. (2025). Impact of preoperative glucagon-like peptide-1 receptor agonist on outcomes following major surgery. World J. Surg..

[30] University of Hong Kong (2024). GLP-1 Receptor Agonist for Reduction of Myocardial Injury After Non-Cardiac Surgery.

[31] Mahmoud A.K., Sheashaa H., Killian M., Awad K., Ibrahim R., Mahmoud A., Farina J., Abdelnabi M., Kamel I., Horn B. (2026). GLP-1 receptor agonists associated with better cardiovascular outcomes in obese or diabetic patients with elevated Lp(a) levels: A Multicenter retrospective study. Int. J. Cardiol..

[32] Kindel T.L., Wang A.Y., Wadhwa A., Schulman A.R., Sharaiha R.Z., Kroh M., Ghanem O.M., Levy S., Joshi G.P., LaMasters T.L. (2025). Multisociety Clinical Practice Guidance for the Safe Use of Glucagon-like Peptide-1 Receptor Agonists in the Perioperative Period. Clin. Gastroenterol. Hepatol..

[33] Perkovic V., Tuttle K.R., Rossing P., Mahaffey K.W., Mann J.F.E., Bakris G., Baeres F.M., Idorn T., Bosch-Traberg H., Lausvig N.L. (2024). Effects of semaglutide on chronic kidney disease in patients with type 2 diabetes (FLOW). N. Engl. J. Med..

[34] Marso S.P., Bain S.C., Consoli A., Eliaschewitz F.G., Jódar E., Leiter L.A., Lingvay I., Rosenstock J., Seufert J., Warren M.L. (2016). Semaglutide and cardiovascular outcomes in patients with type 2 diabetes (SUSTAIN-6). N. Engl. J. Med..

[35] He L., Wang J., Ping F., Yang N., Huang J., Li Y., Xu L., Li W., Zhang H. (2022). Association of glucagon-like peptide-1 receptor agonist use with risk of gallbladder and biliary diseases: A systematic review and meta-analysis of randomized clinical trials. JAMA Intern. Med..

[36] Bjerre Knudsen L., Madsen L.W., Andersen S., Almholt K., de Boer A.S., Drucker D.J., Gotfredsen C., Egerod F.L., Hegelund A.C., Jacobsen H. (2010). Glucagon-like peptide-1 receptor agonists activate rodent thyroid C-cells causing calcitonin release and C-cell proliferation. Endocrinology.

[37] Newsome P.N., Buchholtz K., Cusi K., Linder M., Okanoue T., Ratziu V., Sanyal A.J., Sejling A.-S., Harrison S.A. (2021). A placebo-controlled trial of subcutaneous semaglutide in nonalcoholic steatohepatitis. N. Engl. J. Med..

[38] Wilding J.P.H., Batterham R.L., Calanna S., Davies M., Van Gaal L.F., Lingvay I., McGowan B.M., Rosenstock J., Tran M.T., Wadden T.A. (2021). Once-weekly semaglutide in adults with overweight or obesity (STEP 1). N. Engl. J. Med..

[39] Loganathan J., Cohen A.C., Kaloupis G.M., Harris C., Chronopoulos A., James V., Hamilton J., Green S., Wallis A., Morgan S. (2022). A pilot clinical study to Evaluate Liraglutide-mediated Anti-platelet activity in patients with type-2 Diabetes (ELAID study). J. Diabetes Complicat..

[40] Cameron-Vendrig A., Reheman A., Siraj M.A., Xu X.R., Wang Y., Lei X., Afroze T., Shikatani E., El-Mounayri O., Noyan H. (2016). Glucagon-like peptide 1 receptor activation attenuates platelet aggregation and thrombosis. Diabetes.

[41] Cahill K.N., Amin T., Boutaud O., Printz R., Newcomb D.C., Foer D., Hodson D.J., Broichhagen J., Beckman J.A., Yu C. (2022). Glucagon-like peptide-1 receptor regulates thromboxane-induced human platelet activation. JACC Basic Transl. Sci..

[42] Margulies K.B., Hernandez A.F., Redfield M.M., Givertz M.M., Oliveira G.H., Cole R., Mann D.L., Whellan D.J., Kiernan M.S., Felker G.M. (2016). Effects of liraglutide on clinical stability among patients with advanced heart failure and reduced ejection fraction: A randomized clinical trial (FIGHT). JAMA.

[43] Jorsal A., Kistorp C., Holmager P., Tougaard R.S., Nielsen R., Hänselmann A., Nilsson B., Møller J.E., Hjort J., Rasmussen J. (2017). Effect of liraglutide, a glucagon-like peptide-1 analogue, on left ventricular function in stable chronic heart failure patients with and without diabetes (LIVE)—A Multicentre, Double-Blind, Randomised, Placebo-Controlled Trial. Eur. J. Heart Fail..

[44] Newell P., Asokan S., Zogg C., Prasanna A., Hirji S., Harloff M., Kerolos M., Kaneko T. (2024). Contemporary socioeconomic-based disparities in cardiac surgery: Are we closing the disparities gap?. J. Thorac. Cardiovasc. Surg..

[45] Epic Health Research Network Epic Cosmos Data Platform. https://cosmos.epic.com.

[46] Chiari P., Fellahi J.L. (2024). Myocardial protection in cardiac surgery: A comprehensive review of current therapies and future cardioprotective strategies. Front. Med..

